# Heterospecific social behavior in primates: insights into the evolutionary roots of pet-keeping

**DOI:** 10.1007/s10329-026-01281-0

**Published:** 2026-07-21

**Authors:** Cyril C. Grueter, Fany Roncin, K. Anne-Isola Nekaris, Jill Pruetz, Richard McFarland, Sabit Hasan, Malgorzata Arlet, Timothy M. Eppley, Susan M. Cheyne, Vincent Nijman, Eckhard W. Heymann, Pascal R. Marty, Chiara De Gregorio, Walter Akankwasa, Lakshani Weerasekara, Jerome Micheletta, Ikki Matsuda, Luciana I. Oklander, M. Tarik Kabir, Nicolas Gorostiaga, Chengfeng Wu, Lyna Rachid Martin, Sabina Koirala, Annette Lanjouw, Hassan al Razi, Nicholas Chapoy, Laura M. Bolt, Ian C. Colquhoun, Yanpeng Li, Zhipang Huang, Colin A. Chapman, Paul A. Garber

**Affiliations:** 1 Institute of Human Sciences, School of Anthropology and Museum Ethnography, University of Oxford, Oxford, UK; 2 International Centre for Biodiversity and Primate Conservation, Dali University, Dali, Yunnan China; 3 Department of Anatomy, Physiology and Human Biology, School of Human Sciences, The University of Western Australia, Perth, WA Australia; 4 Centre for Evolutionary Biology, School of Biological Sciences, The University of Western Australia, Perth, WA Australia; 5 Centre of Excellence in Biodiversity and Natural Resource Management, University of Rwanda, Kigali, Rwanda; 6 École Normale Supérieure de Lyon, University of Lyon, Lyon, France; 7 Faculty of Science and Engineering, Anglia Ruskin University, School of Life Sciences, Cambridge, UK; 8 Department of Anthropology, Texas State University, San Marcos, TX USA; 9 NTU Psychology, Nottingham Trent University, Nottingham, UK; 10 Applied Behavioural Ecology and Ecosystems Research Unit, University of South Africa, Florida, South Africa; 11 Department of Anthropology, The University of Texas at San Antonio, San Antonio, TX USA; 12 Faculty of Biology, Institute of Human Biology and Evolution, Adam Mickiewicz University, Poznań, Poland; 13 Wildlife Madagascar, San Diego, CA USA; 14 Department of Anthropology, Portland State University, Portland, USA; 15 Oxford Brookes University, Oxford, UK; 16 Borneo Nature Foundation International, Palangkaraya, Central Kalimantan Indonesia; 17 Verhaltensökologie & Soziobiologie, Deutsches Primatenzentrum – Leibniz-Institut für Primatenforschung, Göttingen, Germany; 18 Wildlife Park Goldau, Goldau, Switzerland; 19 Department of Psychology, University of Warwick, Coventry, UK; 20 Bugoma Primate Conservation Project, Bugoma Central Forest Reserve, Hoima, Uganda; 21 Department of Zoology, Faculty of Science, University of Peradeniya, Kandy, Sri Lanka; 22 Centre for Comparative and Evolutionary Psychology, School of Psychology, Sport and Health Sciences, University of Portsmouth, Portsmouth, UK; 23 Wildlife Research Center, Kyoto University, Sakyo, Kyoto Japan; 24 Chubu University, Kasugai, Aichi Japan; 25 Institute for Tropical Biology and Conservation, Universiti Malaysia Sabah, Kota Kinabalu, Sabah, Malaysia; 26 Grupo de Investigación en Genética Aplicada, Instituto de Biología Subtropical, Consejo Nacional de Investigaciones Científicas y Técnicas (CONICET-UNAM), Posadas, Misiones Argentina; 27 School of Biological Sciences, Universiti Sains Malaysia, George Town, Penang, Malaysia; 28 School of Life Sciences, Sun Yat-Sen University, Guangzhou, Guangdong China; 29 Réserve Africaine de Sigean, Sigean, France; 30 Key Laboratory of Ecological Safety and Sustainable Development in Arid Lands, Xinjiang Institute of Ecology and Geography, Chinese Academy of Sciences, Urumqi, China; 31 Arcus Foundation, New York, NY USA; 32 Department of Anthropology, Tulane University, New Orleans, USA; 33 John H. Daniels Faculty of Architecture, Landscape, and Design, Institute of Forestry and Conservation, University of Toronto, Toronto, ON Canada; 34 Department of Anthropology, The University of Western Ontario, London, Canada; 35 Institute of Eastern-Himalaya Biodiversity Research, Dali University, Dali, Yunnan China; 36 Center for Interdisciplinary Sciences, Dali University, Dali, Yunnan China; 37 Biology Department, Vancouver Island University, Nanaimo, BC Canada; 38 School of Life Sciences, University of KwaZulu-Natal, Scottsville, Pietermaritzburg South Africa; 39 Shaanxi Key Laboratory for Animal Conservation, School of Life Sciences, Northwest University, Xi’an, China; 40 Department of Anthropology and Program in Ecology, Evolution, and Conservation Biology, University of Illinois, Urbana, IL USA

**Keywords:** Heterospecific social behaviour, Primate sociality, Interspecific interactions pet-keeping evolution, Affiliative behavior

## Abstract

Primate interspecific interactions with both other primates and non-primate species range from predator–prey dynamics to affiliative behaviors such as grooming, play, and carrying. While many primates participate in interspecific associations or commensal relationships, to our knowledge interspecific social interactions have never been systematically synthesized. Here, we present the first comprehensive review of such interactions, compiling 427 cases from the literature, media sources, and a global survey of primatologists. These involved 88 primate species and 127 non-primate species (including mammals, birds, reptiles, amphibians, malacostracans, and insects) across wild (n = 311) and captive (n = 111) contexts. The most frequent interactions occurred within or between primate families (e.g., Cercopithecidae–Cercopithecidae, Cebidae–Atelidae), though affiliative behaviors were also directed toward distantly related taxa such as dogs and birds. Play (n = 139) and grooming (n = 136) were most common. Juveniles and infants predominantly engaged in play, while adult females were more likely to groom. Adult males were less affiliative overall. Contrary to expectations, affiliative behaviors were not more often directed toward immature than adult heterospecifics. Primates were the initiators in most interactions. These findings offer new insights into the diversity and contexts of heterospecific sociality in primates and suggest that some behavioral tendencies relevant to later forms of human–animal companionship – such as caregiving, tolerance, and exploratory play – may be more widespread among primates than previously recognized.

**Supplementary Information:**

The online version contains supplementary material available at https://doi.org/10.1007/s10329-026-01281-0.

## Introduction

Throughout human history, animals and their parts have played a vital role in human subsistence as food, weapons, textiles and ornaments (Richards & Trinkaus 2009; Alves et al. 2018; Baldia & Armitage 2023; de la Torre et al. 2025). At least 17,000 years ago, humans began to domesticate wild animals for a diversity of economic and evolutionary purposes (Vigne 2011), including for food, dress and accessories, domestic textiles, weapons and armour, guardianship and protection (Scanes 2018; Kayabasi & Yılmaz 2022). Out of such relationships has emerged pet ownership, which Eddy (2003) defines as an animal kept for pleasure rather than utility (although some animals may simultaneously provide both utility and companionship). Importantly, pet keeping is not a uniquely modern phenomenon, but is attested in the archaeological record through evidence of prolonged human–animal care and intentional burial, particularly of dogs, suggesting that companion-like relationships have deep historical roots with potential evolutionary significance (Serpell 1996).

Although a human guardian may expend significant resources on another species for no obvious material gain, pet keeping has been linked to potential evolutionary fitness gains such as improved physical fitness, social connections, stress relief and companionship (Archer 1997; Walsh 2009; Pongrácz & Dobos 2023). But is pet keeping uniquely human? To understand the origin of this behaviour amongst humans, we sought to explore interspecific interactions amongst primates to distinguish characteristics of their interactions with other species that might have selected for pet keeping in humans.

Primates engage in a wide range of interspecific interactions, with both primate and non-primate species. These interactions can include predator–prey dynamics (Hausfater 1976; Stanford et al. 1994) and competitive encounters involving aggression or displacement, particularly over valuable resources (Morris & Goodall 1977; Falótico et al. 2021). Primates also form non-aggressive associations with a variety of species. A substantial number of primate species participate in *mixed-species associations*, which involve coordinated behavior and sustained proximity between two or more – often taxonomically close – species (Cords 2000; Rehg 2016). Similarly, typically shorter and more opportunistic encounters, often centered on shared resource patches, have also been documented across various taxa (Stensland et al. 2003; Waser 1982). Both types of associations are most commonly interpreted as providing foraging advantages and/or antipredator benefits to all species participating (Struhsaker 1981; Terborgh 1983; Cords 1990; Stensland et al. 2003). In contrast, *primate–non-primate associations* appear to be commensal, with non-primates gaining foraging advantages such as access to dropped fruit, leaves, or flushed prey, and anti-predator benefits from primate alarm calls and vigilance (e.g. Havmøller et al. 2021). In such associations, primates typically receive little to no benefit, making true mutualism rare (Heymann & Hsia 2015).

Primates engage in social interactions not only with other primates but also with non-primate species (including, but not limited to, mammals from various orders, birds, and reptiles) across a range of contexts, including captivity, anthropogenically disturbed, and more pristine habitats (Ross et al. 2009; Hockings et al. 2012; Sanz et al. 2022). In the wild, such interspecific interactions may arise in the context of mixed-species associations (e.g., Waser 1980; Struhsaker 1981) but they are by no means restricted to these ecological arrangements. A fundamental aspect of pet keeping among humans is either playing with or grooming/stroking their pets (Rooney et al. 2001; Henning et al. 2022). Interestingly, among the most frequently observed non-agonistic interspecific social behaviours are allogrooming (e.g., Ihobe 1990; Rose et al. 2003; Eppley et al. 2015; Freymann et al. 2023; Kabir et al. 2023; Galotti et al. 2024) and play (e.g., Struhsaker 1975; de Resende et al. 2004; Hockings et al. 2012; Sanz et al. 2022; Brooks & Burghardt 2023). Many of the benefits attributed to allogrooming in intraspecific interactions – such as hygiene maintenance, thermoregulation and the formation or reinforcement of social bonds (Dunbar 1991; Zamma 2002a; Grueter et al. 2013; McFarland et al. 2016) – may also apply to grooming across species boundaries. Similarly, the key developmental functions of play in conspecific contexts, i.e., improvements in motor coordination and the development of social and cognitive skills, are likely to extend to interspecific play among immature individuals of different species (Poirier & Smith 1974; Fagen 1981; Spinka et al. 2001; Heintz et al. 2017; Richardson et al. 2025).

Other documented affiliative behaviors between primates and heterospecifics include huddling (Eppley et al. 2015; Law et al. 2021), carrying or being carried (Ziegler 2002; Eppley et al. 2015; Yokoyama 2021), food sharing (Pearson et al. 2010; de Carvalho Oliveira et al. 2017), nursing (Izar et al. 2006), and mounting (Pelé et al. 2017; Ogawa et al. 2019; Sanz et al. 2022). Because such interactions may be more unpredictable and potentially riskier than those with conspecifics – and can, in some cases, escalate into abusive behaviour, such as abductions and physical maltreatment (Goldsborough et al. 2025; Hockings et al. 2012; Pi et al. 1993; Zamma 2002b) – they are expected to be uncommon.

Despite a growing body of anecdotal reports, to our knowledge interspecific social interactions involving nonhuman primates have never been systematically synthesized. Here, we provide the first comprehensive review of social (in particular affiliative/socio-positive) interactions between primate species and between primates and non-primate species across a range of ecological contexts, including both captive and wild settings. This project fills a critical gap in our understanding by compiling and analyzing existing knowledge on these interactions. The primary objective is to identify which species are most frequently involved in heterospecific social behavior and to determine some of the ecological and social conditions under which these interactions occur. To maintain a focused scope, we explicitly exclude human–nonhuman animal interactions such as pet-keeping or companion animal situations (Olmert 2009; Melson 2019).

Pet keeping in humans is diverse and not limited to companionship alone, but may also involve caregiving, fascination with unusual or aesthetically appealing animals, novelty seeking, symbolic value, or status display. Motivations for maintaining close relationships with other species can therefore vary substantially across individuals and cultures, and may differ between men and women (Herzog 2007; Gray & Young 2011). In this broader comparative sense, heterospecific social interactions in nonhuman primates may illuminate some of the behavioral foundations relevant to later forms of human–animal companionship and animal keeping. These include exploratory play, tolerance toward other species, caregiving tendencies, and selective affiliative responses toward particular heterospecifics. Importantly, our hypotheses are grounded primarily in primate behavioral ecology rather than direct analogies with modern human pet keeping, although they may nevertheless help clarify behavioral predispositions that facilitated the emergence of long-term human–animal relationships.

Given the scarcity of systematic data, our primary intent is to provide a general overview of the types of heterospecific interactions noted in published manuscripts, survey responses, and online media. Secondarily, we also conduct tests of a few preliminary hypotheses. First, we hypothesize that heterospecific social interactions are more common between phylogenetically closely related species, which are more likely to occur in close geographic proximity, share ecological similarities (e.g., habitat requirements and diet), and possess comparable communicative signals and behavioral repertoires, thereby facilitating mutual understanding and affiliative engagement. Second, we evaluate the hypothesis that female primates – due to their predominant role as caregivers in most species – are more likely than males to exhibit heterospecific nurturing behaviors. Third, we predict that interspecific play will occur more frequently among juveniles, consistent with intraspecific patterns in primates and other mammals (Fagen 1981). Because play is often exploratory and socially flexible, juvenile interspecific play may also represent an important developmental context in which tolerance and engagement toward heterospecifics emerge. Fourth, at a more proximate level, we predict that immature heterospecifics are preferred as interaction partners, likely because their infantile physical features elicit caregiving or affiliative responses via evolved perceptual biases (Lorenz 1943; Kawaguchi et al. 2019). Finally, we explore whether some of these interspecific relationships are functionally or structurally analogous to ‘pet-keeping’, a behavior traditionally regarded as uniquely human. Our review provides a foundation for considering whether some interspecific interactions by nonhuman primates provide the basis for rudimentary forms of pet-keeping, thereby offering new insights into the evolutionary roots of interspecies bonding.

## Materials and methods

### Data collection

To compile a comprehensive dataset on interspecific social interactions involving primates, we employed a multi-pronged data collection strategy combining systematic literature searches, media screening, and a standardized online survey.

We conducted an extensive literature review using search terms such as “affiliative or friendly” AND “primates” AND “interspecific interactions” across major scientific databases, i.e., Web of Science and Google Scholar. In addition, we searched for visual evidence by screening publicly available videos and images through platforms such as Google Images, Google Video, and YouTube. Search terms included those used in the literature review, as well as additional phrases such as “unusual animal friends” and “animal odd couples.” These searches aimed to capture both scientific and popular representations of affiliative interspecific interactions involving primates. To improve reliability, we applied several screening criteria to media-derived observations. We only included cases in which the species involved and the behavioral interaction could be confidently identified from the visual material and accompanying contextual information. Videos or images that appeared staged, heavily edited, ambiguous, duplicated across sources or lacking sufficient contextual detail were excluded. Media-based records were used primarily to broaden the descriptive scope of the dataset and to document rare interaction types that may be underrepresented in the formal literature. To reduce duplicate reporting across source types, we cross-checked observations using species identities, geographic locations, behavioral descriptions, contextual details, and, where available, the timing of observations. Very few potential duplicate records were identified, and records judged to refer to the same interaction event were consolidated or excluded.

To collect standardized data on observed interspecific social interactions, we designed an online questionnaire using Qualtrics. The survey included questions on the observer’s identity, primate and non-primate species involved, geographic location, age and sex of individuals, behavior type, direction and initiation of the interaction, environmental context, duration of interaction, and any injury outcomes.

The questionnaire was distributed to approximately 250 researchers via email. Targeted recruitment was conducted by reviewing all primate field research articles published in the past six years in three leading journals: *American Journal of Primatology*, *International Journal of Primatology*, and *Primates*. Where available, the contact details of corresponding authors were used for survey distribution. Additional contacts were identified through professional networks (including colleagues of C. Grueter) and researchers working in zoological institutions. A total of 37 researchers responded to the survey.

### Data analysis

Our curated dataset of heterospecific social interactions involving primates comprised a total of 427 observations: 303 sourced from the scientific literature, 58 from media (photos and videos), and 66 from survey responses (Table S1). The dataset therefore combines peer-reviewed publications, survey responses, and screened media observations (see Supplementary Table S1 for the classification of individual records). Each entry included information on the primary actor (species, age, sex), the recipient (species, age, sex), the type and subtype of behavior performed (see below), the direction of the interaction, and the context (wild or captive). For the statistical models, we restricted the dataset to entries in which both the age and sex of the primary individual were recorded. Subadult individuals were grouped with adults, while juveniles and infants were retained but not subdivided by sex because their sex was often not reported or could not be reliably determined. Age-sex categories were defined as: "Adult Female" (reference), "Adult Male", "Juvenile", and "Infant".

Behaviors were grouped into three overarching types: affiliative, abusive, and sexual. Subtypes included: body contact, carry, close proximity, copulate, greet, groom, hold, huddle, mount, nurse, play, ride on back, share food, solicit mating, suckle, touch, and treat abusively. Subtypes with very low frequencies, i.e., “suckle,” “solicit mating,” “copulate,” and “nurse” were excluded from statistical analyses. In the statistical models, we focused on two comparisons: (1) Affiliative vs. Abusive behavior types, and (2) the two most commonly observed subtypes, “groom” and “play”.

Statistical analyses were conducted in R (version 4.4.0; R Core Team, 2025). We used generalized linear models (GLMs) to examine the effect of age–sex class on the probability of different types of interspecific interactions. Models were fitted with the function glm(), specifying a binomial error distribution and a logit link function suitable for binary response variables. Two models were constructed: Model 1 compared affiliative versus abusive behavior types (coded as 1 = affiliative, 0 = abusive; N = 237), and Model 2 compared grooming versus play interactions (coded as 1 = grooming, 0 = play; N = 167). In both models, age–sex class was included as a categorical predictor, with “adult female” specified as the reference category. To test whether affiliative interactions by adults of the primary species were more likely to be directed toward immature (infant or juvenile) versus adult individuals of another species, we used an exact binomial test.

### Ethical considerations

The study protocol was reviewed and approved by the Human Research Ethics Committee of the University of Western Australia (2025/ET000232), ensuring compliance with guidelines for informed consent, data protection, and voluntary participation.

## Results

The 427 recorded interspecific social interactions involved 88 unique primary species (all primates) and 127 unique partner species (72 primates, 55 non-primates), resulting in 204 distinct species pairings. These interactions were categorized into 17 behavioral subtypes, with play (n = 139) and grooming (n = 136) being the most frequently observed. Contextual data were available for most cases: 310 interactions occurred in wild settings and 110 in captive environments. Among the wild interactions, the vast majority (90.3%) involved wild heterospecifics, of which 67% were other primates. The remainder featured domesticated species in proximity to human habitation, including dogs (*Canis familiaris*; n = 26), pigs (*Sus scrofa domesticus*; n = 2), and cats (*Felis catus*; n = 2).

Of all the interspecific social interactions compiled, directionality was reported in 219 cases. Most were primate-initiated (144; 66%), followed by bidirectional interactions (72; 33%), and only 3 cases (< 1%) were initiated by non-primates. Direction was unreported in 206 cases (48%). Grooming was primarily primate-initiated, while play was often bidirectional. Carrying behavior was mostly initiated by primates, but included all three non-primate-initiated cases: two by dogs and one by a lion (*Panthera leo*).

The most frequent interaction pairs between primary and other species observed under human care and in the wild are shown separately in Tables S2 and S3, respectively. In the wild, the most frequent interaction was between Panamanian white-faced capuchins (*Cebus imitator*) and black-handed spider monkey (*Ateles geoffroyi*; n = 19), followed by Japanese macaques (*Macaca fuscata*) with Sika deer (*Cervus nippon*; n = 12), chimpanzees (*Pan troglodytes*) with red-tailed monkeys (*Cercopithecus ascanius*; n = 11), and rhesus macaques (*Macaca mulatta*) with domestic dogs (n = 11).

Most primary species belonged to the family Cercopithecidae (n = 211), followed by Hominidae (n = 87), Cebidae (n = 46), and Callitrichidae (n = 29). Other represented families included Atelidae (n = 20), Lemuridae (n = 17), Hylobatidae (n = 8), Aotidae (n = 2), Lorisidae (n = 2), Pitheciidae (n = 1), Galagidae (n = 1), and Indriidae (n = 1).

**Fig. 1:**
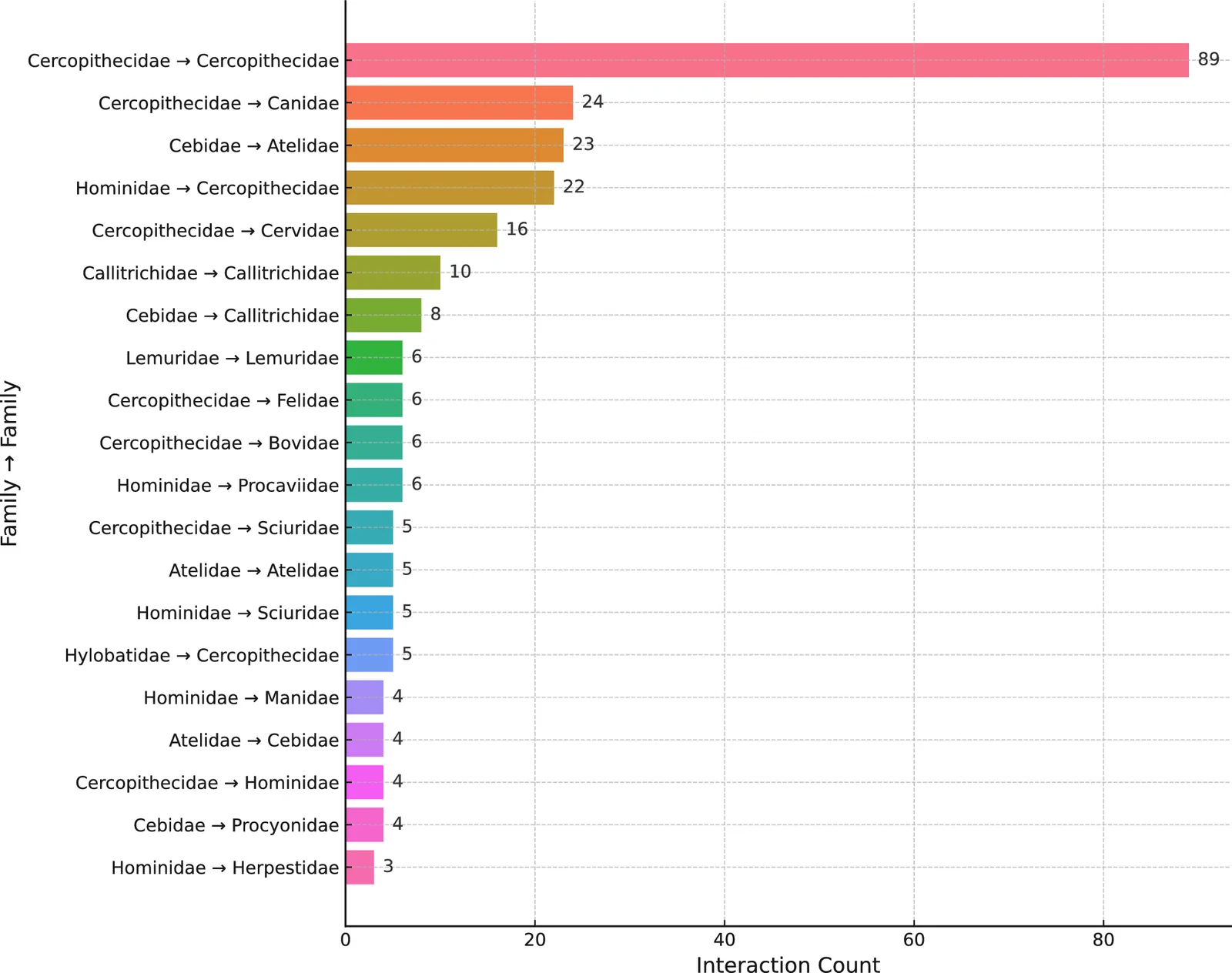
Top 20 most frequent interspecific social interactions in the wild, aggregated at the family level. Each bar represents the number of recorded interactions in which the primary species belonged to the family listed on the left and the other species to the family on the right

**Fig. 2:**
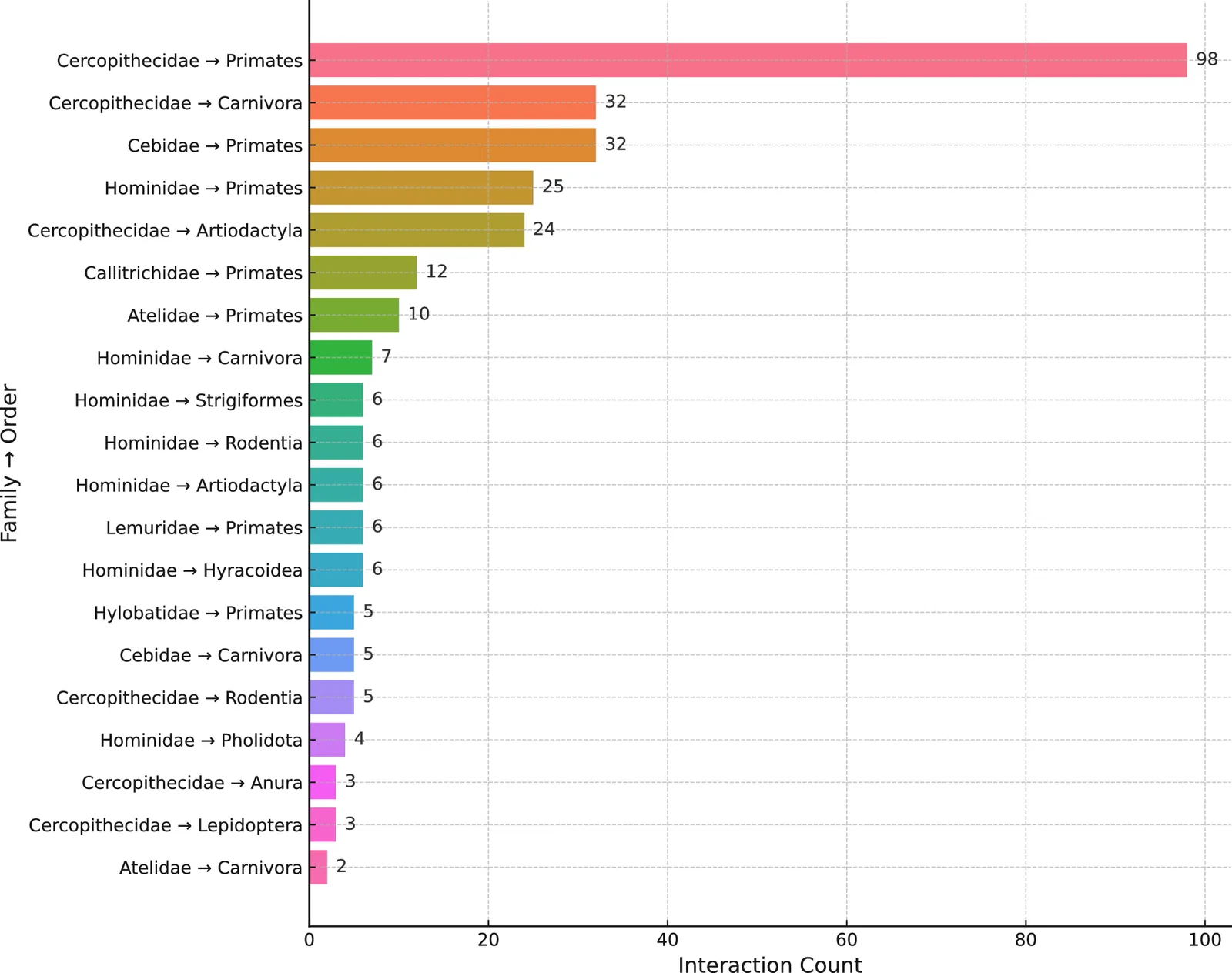
Top 20 most frequent interspecific social interactions in the wild, aggregated by the family of the primary species and the order of the other species. Each bar represents the number of observed interactions involving a primate from the specified family and a non-conspecific species from the corresponding order

**Fig. 3:**
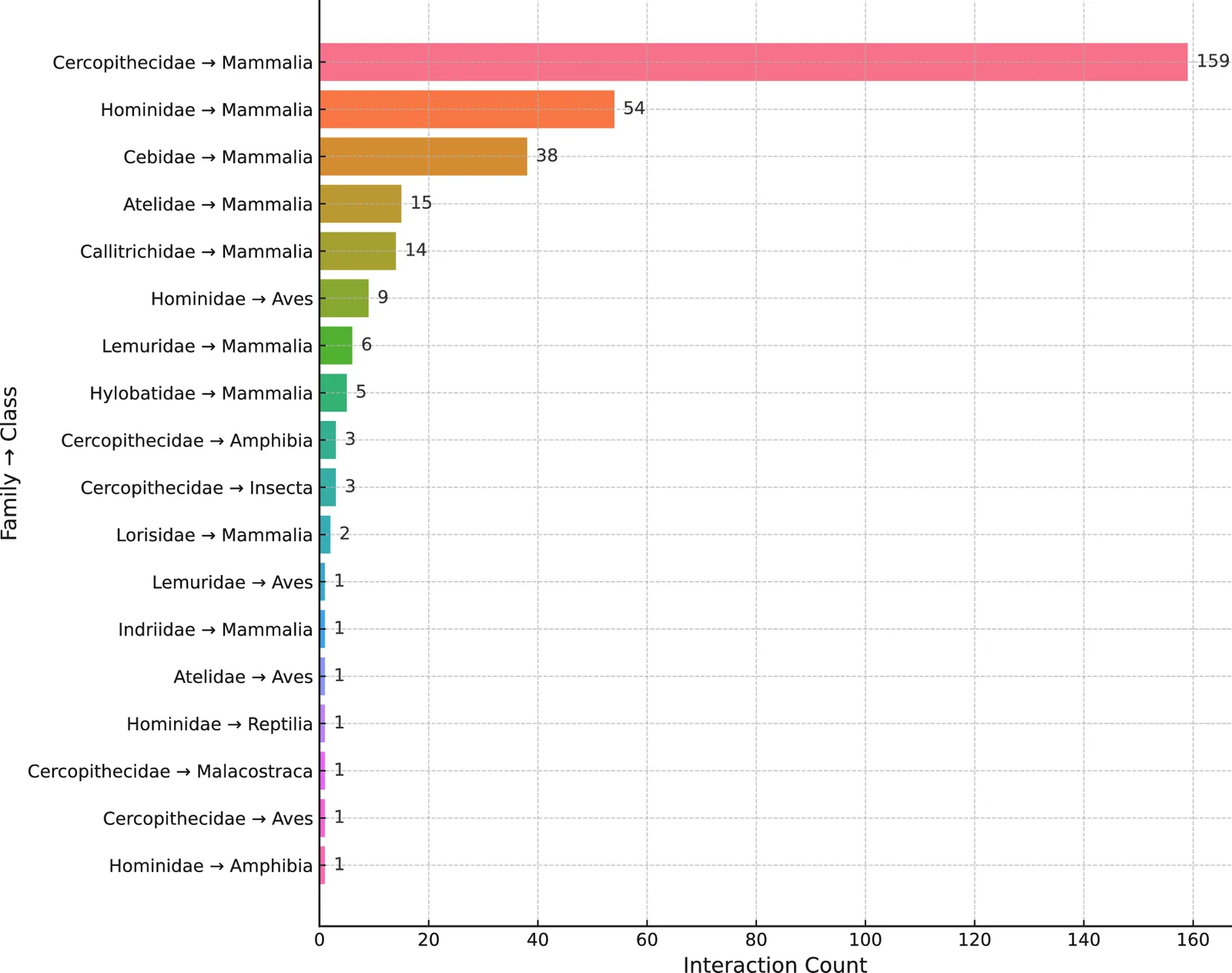
Top 20 most frequent interspecific social interactions in the wild, summarized by the family of the primary species and the class of the other species involved. Bars represent the number of observed interactions between primates and non-conspecific species from various animal classes

At the Family level, the most frequent interspecific interactions in the wild occurred between members of the family Cercopithecidae, followed by interactions between Cercopithecidae and Canidae, Cebidae and Atelidae, Hominidae and Cercopithecidae and Cercopithecidae and Cervidae (Fig. 1; Table S4). When grouped by the Order of the interacting species, the most frequent heterospecific interactions in wild contexts involved primates from the family Cercopithecidae interacting with members of the Order Primates. This was followed by Cercopithecidae–Carnivora, Cebidae–Primates, Hominidae–Primates, and Cercopithecidae–Artiodactyla pairings (Fig. 2). When grouped by the Class of the interacting species, the five most frequent pairings were between Cercopithecidae and Mammalia, Hominidae and Mammalia, Cebidae and Mammalia, Atelidae and Mammalia, and Callitrichidae and Mammalia. Interactions with other classes such as Aves, Amphibia, Reptilia, Insecta, and Malacostraca were less common (Fig. 3).

Of the 26 recorded abusive interactions directed by primates, the vast majority (21) involved non-primate species, while only five were directed at other primates. Most targets belonged to the class Mammalia (n = 18), including rodents, carnivores, pangolins, hyraxes, and suids. Additional targets included species from Aves (n = 4), Amphibia (n = 2), and Insecta (n = 2). Although we were unable to collect a large, systematic sample on the outcomes of these interactions, we documented 13 confirmed cases of death resulting from violent handling of heterospecifics. These fatalities included seven birds, two pangolins, one domestic cat, and three primates.

**Fig. 4:**
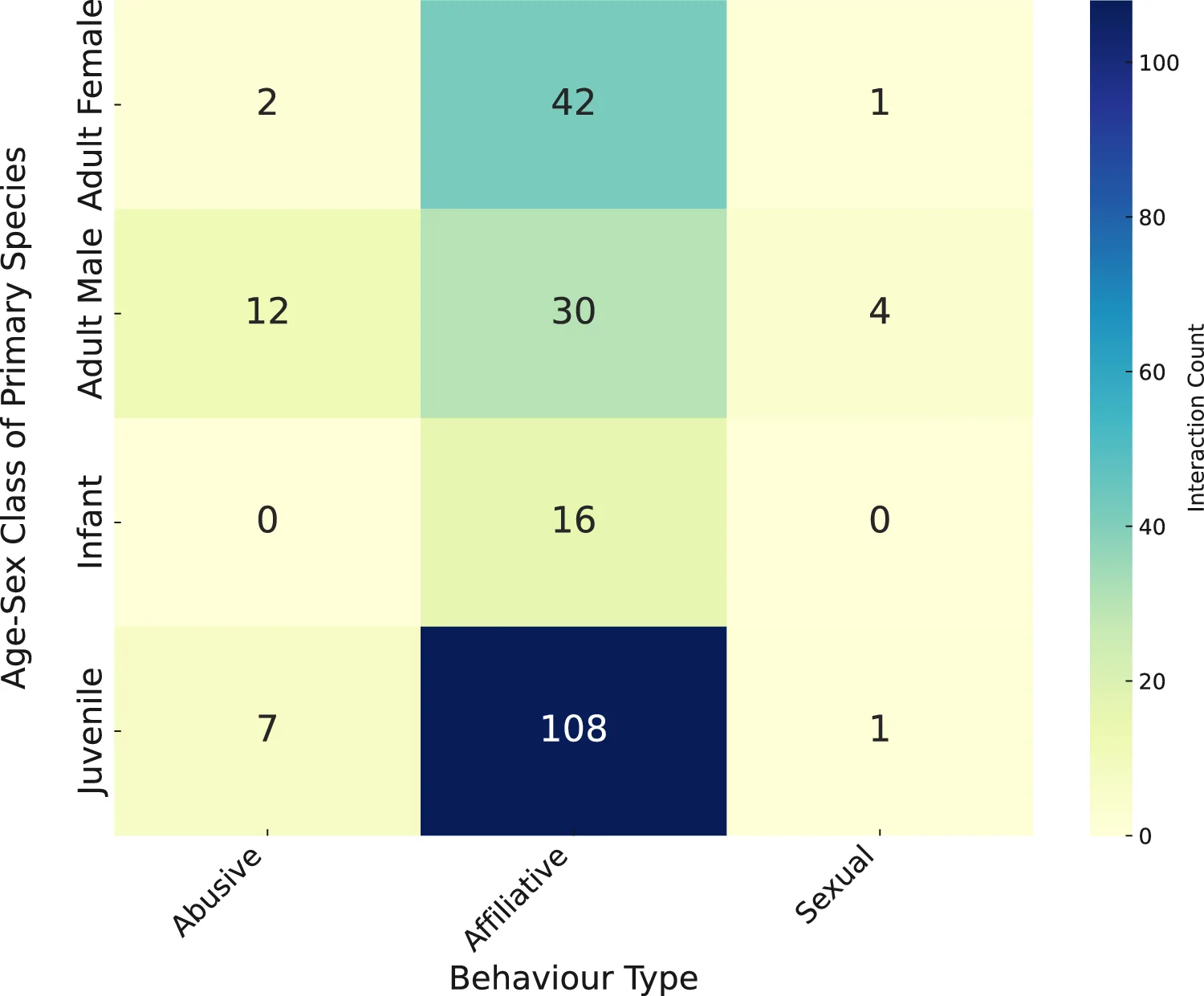
Frequency matrix of interspecific social interactions by behavioral type and age–sex class of the primary species. Each cell shows the number of recorded interactions for a specific behavioral type (abusive, affiliative, sexual) initiated by a particular age–sex class (adult male, adult female, juvenile, infant)

**Fig. 5:**
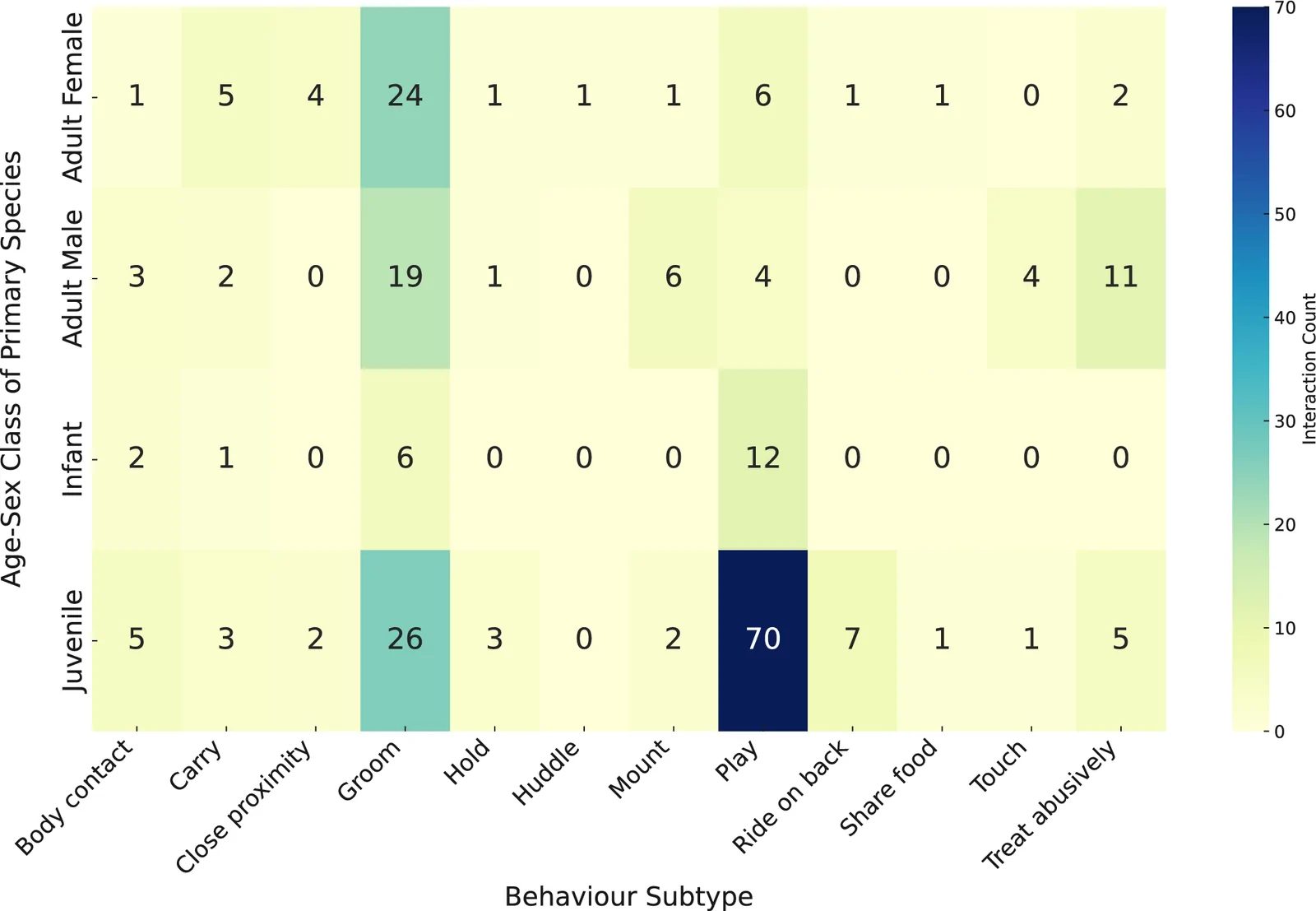
Frequency matrix of interspecific social interactions by behavioral subtype and age–sex class of the primary species. Each cell shows the number of recorded interactions for a specific behavioral subtype (e.g., grooming, playing, food sharing) initiated by a particular age–sex class (adult male, adult female, juvenile, infant)

Adult males were significantly less likely than adult females to engage in affiliative behavior (Table 1; Fig. 4). Predicted probabilities indicated that adult females had the highest likelihood of affiliative interaction, followed by juveniles, with adult males substantially lower. Juveniles and infants were significantly more likely to engage in play rather than grooming (Table 2; Fig. 5). Predicted probabilities showed that adult females had the highest likelihood of grooming, while juveniles and infants overwhelmingly play.

**Table 1:** Results of binomial GLM predicting the likelihood of affiliative (vs. abusive) interspecific behavior as a function of age–sex class

Predictor	Coefficient	Std. error	z-value	p-value	95% CI
Adult male	−1.72	0.61	−2.8	0.005	[−2.91, −0.53]
Juvenile	−0.44	0.59	−0.76	0.452	[−1.60, 0.72]
Infant	−1.19	0.74	−1.61	0.105	[−2.64, 0.26]

**Table 2:** Results of Model 2, examining the effect of age–sex class on the probability of a grooming (vs. play) interaction using a binomial GLM

Predictor	Coefficient	Std. error	z-value	p-value	95% CI
Adult male	0.17	0.72	0.24	0.81	[−1.24, 1.59]
Juvenile	−2.38	0.54	−4.42	< 0.001	[−3.44, -1.32]
Infant	−2.08	0.66	−3.14	0.002	[−3.37, -0.78]

We also examined whether affiliative interactions by adults were more likely to be directed toward immature (infant or juvenile) versus adult individuals of other species. Among all cases with age information for the recipient (n = 81), we found no statistically significant difference: 44 interactions were directed toward adults and 37 toward immatures (binomial test, p = 0.505). However, when restricting the sample to cases in which the primary actor was an adult female (n = 34), affiliative interactions were significantly more likely to be directed toward adults (25 adults vs. 9 immatures; binomial test, p = 0.009).

**Fig. 6:**
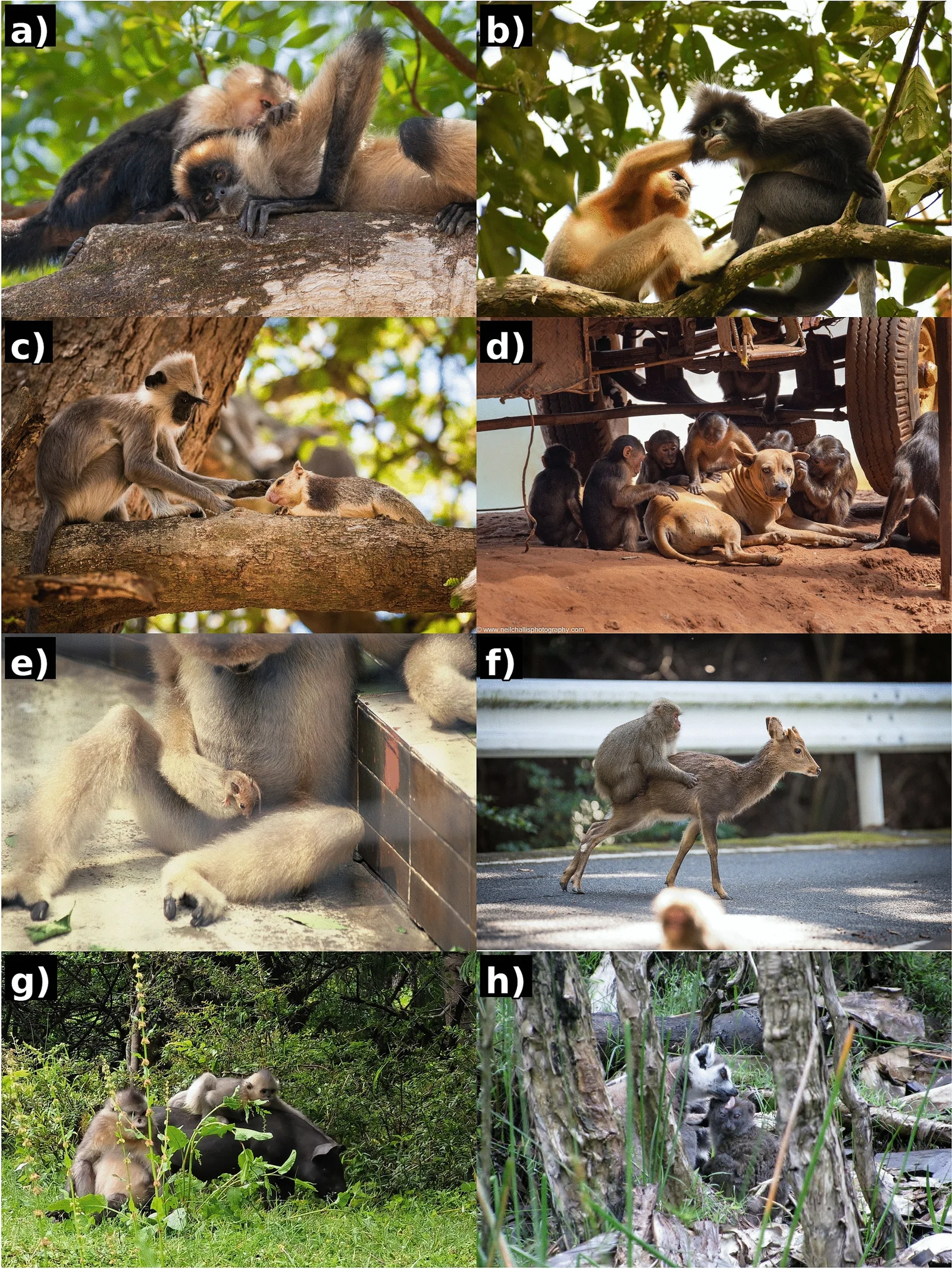
Examples of affiliative interspecific interactions involving non-human primates. **a** Adult female white-faced capuchin (*Cebus imitator*) monkey grooming an adult female black-handed spider monkey (*Ateles geoffroyi*) in Santa Rosa, Costa Rica; photo by Nicholas Chapoy. **b** Capped langur (*Trachypithecus pileatus*) grooming a Phayre's leaf monkey (*Trachypithecus phayrei*) in Satchari National Park Bangladesh; photo by Sabit Hasan. **c** A tufted gray langur (*Semnopithecus priam*) stroking a grizzled giant squirrel (*Ratufa macroura*) in Yala National Park, Sri Lanka; photo by Senthi Aathavan Senthilverl, via Wikimedia Commons, licensed under CC BY-SA 4.0. **d** Stump-tailed macaques (*Macaca arctoides*) grooming a domestic dog at Khao Tao Mo temple, Thailand; photo by Neil Challis. **e** Female northern white-cheeked gibbon (*Nomascus leucogenys*) holding a mouse at the Shanghai Zoo, China; photo by Cyril Grueter. **f** Japanese macaque (*Macaca fuscata*) mounting and riding on the back of a sika deer (*Cervus nippon*) at Yakushima, Japan; photo by Alexandre Bonnefoy and Cédric Sueur. **g** Young juvenile black-and-white snub-nosed monkey (*Rhinopithecus bieti*) playing on a domestic pig at Xiangguqing, Yunnan Golden Monkey National Park, China; photo by Yanpeng Li. h) Ring-tailed lemur (*Lemur catta*) grooming a southern bamboo lemur (*Hapalemur meridionalis*) in Mandena, Madagascar; photo by Timothy Eppley

Figure 6 shows examples of affiliative interspecific interactions involving primates.

## Discussion

### Ecological and phylogenetic correlates of heterospecific social interactions

In this study, we found a substantial number of primate heterospecific social interactions, many of which were affiliative and may illuminate behavioral tendencies potentially relevant to the evolutionary roots of human–animal companionship and pet keeping. The most frequent interspecific interactions in our dataset involved species from the families Cercopithecidae, Hominidae, and Cebidae, with white-faced capuchins and black-handed spider monkeys representing the most observed wild dyad. It is important to note, however, that the taxonomic breadth of our dataset is necessarily influenced by research effort: some species have been observed extensively over decades, whereas others remain scarcely studied. This uneven coverage likely biases the representation of interspecific interactions and should be considered when interpreting patterns across taxa. Rather than treating these observations as isolated curiosities, several recurring behavioral tendencies emerge across taxa and contexts, including exploratory play, caregiving, social tolerance, and affiliative engagement with socially compatible heterospecifics. These recurring patterns may provide a useful comparative framework for considering behavioral predispositions relevant to the evolutionary origins of human–animal companionship.

Interactions were especially frequent between members of the same primate family (such as Cercopithecidae–Cercopithecidae) followed by combinations like Cercopithecidae–Canidae and Cebidae–Atelidae. These patterns suggest that affiliative heterospecific interactions are more likely to occur between phylogenetically closely related species, as predicted by hypothesis 2 although the Cercopithecidae–Canidae interactions do not align with this. Similarly, Brooks & Burghardt (2023) found many observations of inter-specific play consisting of species playing with phylogenetically proximate taxa. Such species are more likely to share similar communicative signals, behavioral repertoires, and social cognitive capacities, which may facilitate mutual understanding and reduce the likelihood of misinterpretation or conflict. In addition, shared evolutionary histories may result in overlapping ecological niches, further increasing opportunities for affiliative engagement.

Importantly, some of the heterospecific social interactions documented here occur within broader ecologically grounded mixed-species associations. In such cases, affiliative behaviors such as grooming or play may emerge in contexts initially shaped by antipredator benefits, foraging opportunities, or spatial proximity rather than motivations directly analogous to human pet keeping. At the same time, other interactions – particularly those involving prolonged caregiving, repeated affiliative engagement, or interactions with domesticated species – may provide more informative parallels for understanding the behavioral foundations of human–animal companionship.

### Affiliative and abusive interactions across species boundaries

While many interactions were clearly affiliative, it was not uncommon for them to include elements of neglect or even abuse. For example, female primates carrying heterospecific infants often exhibited allomaternal behaviors, but in some cases, rough handling or disregard contributed to the infant's death. Richard (1970) recounts an instance in which three female black-handed spider monkey took turns carrying a mantled howler monkey (*Alouatta palliata*) infant, possibly after stealing it. The infant became an object of social interest, with each female attempting to pull it away from the current carrier by its limbs. On one occasion, a female rested for half an hour with the infant pinned beneath her, despite its loud vocalizations. The infant was later found on the ground and died shortly afterward. Comparable patterns of rough handling with fatal outcomes have also been described in cases of corpse-directed play, in which primates interact with deceased infants in ways that appear exploratory or playful rather than overtly aggressive. Such behavior has been documented in chimpanzees and other primates, where individuals manipulate, carry, or play with corpses, sometimes including heteropecifics (e.g., a dead bushbaby), highlighting that lethal consequences may arise from misdirected play or caregiving tendencies rather than predatory intent (Negrey & Langergraber 2020).

We also documented a small number of behaviors that were predominantly, if not exclusively, abusive in nature, e.g., coercive forms of appropriation. One such case involved a male Hamadryas baboon (*Papio hamadryas*) in Ta’if, Saudi Arabia, who appeared to have kidnapped a domestic puppy, carrying it for extended periods (BBC Earth 2015). Among the abusive behaviors documented, the majority were directed at non-primate species, including mammals, birds, amphibians, and insects. This pattern suggests that while primates are capable of forming affiliative interactions or even proto-relationships with a wide range of species (including distantly related ones), aggression appears more likely to be directed toward those with whom they may share fewer communicative or behavioral similarities. Other differences may also contribute, not least the fact that these target species are often smaller than the primates that abuse them. One possible explanation is that empathy and social inhibition are more readily engaged with species that exhibit familiar or interpretable cues (Miralles et al. 2019). However, given the low frequency of abusive interactions overall, these findings should be interpreted cautiously. They nonetheless raise the possibility that phylogenetic proximity may shape not only the likelihood of affiliative engagement but also the directionality of abusive interactions.

### Age and sex differences in heterospecific social behavior

Our findings provide partial support for the three hypotheses proposed regarding sex, age, and partner characteristics in primate heterospecific affiliative behavior. As for the second hypothesis, consistent with the prediction that female primates are more likely than males to exhibit nurturing behaviors across species boundaries, adult females were significantly more likely than adult males to engage in affiliative interactions, supporting the view that caregiving tendencies in female primates generalize beyond conspecifics. This aligns with established patterns in intraspecific primate behavior, where females are typically the primary caregivers (Clutton-Brock 1991).

In contrast, the fourth hypothesis, i.e., that immature heterospecifics may be preferred as recipients of affiliative behavior due to their infantile features and the perceptual biases these elicit, was not supported. Among interactions in which the age of the non-primate partner was known, there was no overall preference for adults versus immatures. Moreover, when the sample was restricted to cases where the primary actor was an adult female, affiliative interactions were significantly more likely to be directed toward adult individuals. These findings suggest that infantile appearance alone is unlikely to constitute a primary trigger for affiliative engagement in interspecific contexts. While some studies suggest that infant stimuli can attract visual attention in non-human primates (Kawaguchi & Waller 2024), the specific effect of the baby schema (particularly in terms of facial features) has not been systematically verified across species and may not generalize to heterospecifics. Instead, factors such as behavioral compatibility, social attentiveness, or mutual tolerance may play a stronger role in shaping these interactions. One possible explanation for the absence of a preference toward immature heterospecifics is that many species involved in these interactions, particularly domesticated or human-associated taxa, retain neotenous traits into adulthood. As a result, affiliative responses may not depend specifically on immature age classes. Additionally, immature individuals may have been less behaviorally or spatially accessible due to maternal protection or social buffering, potentially reducing opportunities for affiliative contact.

The third hypothesis, i.e., that interspecific play would be more common among juvenile individuals, was strongly supported. Juveniles and infants were significantly more likely to engage in play than grooming, while adult females were more likely to engage in grooming. This pattern closely mirrors intraspecific developmental trajectories in primates and other mammals, in which play peaks in immaturity and gives way to more structured affiliative behaviors, such as grooming, in adulthood (Fagen 1981; Palagi 2018). These findings indicate that play, as a relatively low-risk and exploratory social behavior, may extend to heterospecific partners most readily during the juvenile period. In addition to developmental predispositions, naturally occurring demographic effects may also shape patterns of heterospecific play. In small or demographically skewed groups that lack multiple juveniles of similar age or size, immature individuals may be more likely to seek play partners among heterospecifics simply because age- and size-matched conspecific partners are unavailable. Under such conditions, heterospecific play may reflect opportunity structure rather than an intrinsic preference for other species.

Our analysis also reveals that nonhuman primates are the primary initiators of interspecific social interactions, accounting for two-thirds of all cases where direction was known, while interactions initiated by non-primates were exceedingly rare. This directional bias likely reflects primates’ high social motivation, cognitive flexibility, and propensity for exploratory or nurturing behavior. Although nearly half of the cases lacked direction data, the consistent pattern across diverse sources suggests a genuine asymmetry rather than a reporting artifact.

### Environmental context and social opportunity

Of the 427 recorded interactions, 311 occurred in wild settings and 111 in captive environments. Among the wild cases, 9.7% involved domesticated species such as dogs, cats, and pigs, and were typically observed near areas of human activity where primates may encounter and interact with more tame or habituated animals. These interactions often occurred at the interface between natural habitats and human-modified landscapes. Moreover, interspecies associations and affiliative interactions may also emerge as a consequence of severe habitat degradation. In environments where primate populations are fragmented or conspecifics are scarce, individuals may turn to heterospecific partners in the absence of familiar group members (Kabir et al. 2023).

In captivity, heterospecific interactions often arise as a result of spatial constraints, particularly when different species are housed together in mixed-species enclosures. In many cases, such interactions are actively facilitated by keepers or facility managers who deliberately introduce animals of different species into shared spaces to promote enrichment or companionship (Shepherdson 2003). Heterospecific affiliations are especially common in captive settings where individuals may lack access to age-appropriate conspecifics, e.g., in small social groups, or in contexts where primates are hand-raised or kept as pets. The absence of suitable conspecific partners can lead animals to seek social engagement with heterospecific companions. Additionally, animals in human care typically have regular access to food, reducing the need for foraging and freeing up both time and energy for other activities. This relaxed energetic environment may further encourage affiliative interactions – such as play – when opportunities arise (Brooks & Burghardt 2023). A similar argument has been put forward for wild, yet provisioned, primate populations (Izar et al. 2006).

### Implications for understanding the primatological roots of pet keeping in humans

Patterns of heterospecific social interactions in humans and nonhuman primates show both similarities and differences. A notable commonality is the overrepresentation of mammals—followed to a lesser extent by birds—as companions, whereas reptiles, amphibians, fish, and invertebrates are rarely chosen. In humans, this pattern is largely explained by the prevalence of domesticated species among companion animals, the majority of which are mammals. In nonhuman primates, as discussed above, affiliative interactions may be biased toward taxonomically similar species due to shared ecological niches or greater ease of socio-cognitive communication. In contrast to nonhuman primates, however, humans primarily interact with carnivorans—especially cats and dogs—which reflects their central role in the history of domestication.

Our comparative analysis revealed that female primates are more likely than males to exhibit socio-positive behaviors toward individuals of other species. This pattern aligns with ethnographic reports from hunter-gatherer societies, where women have been documented caring for (and in some cases even breastfeeding) a wide range of animal species (Erikson 2000; Serpell 1989). A meta-analysis on gender differences in human–animal interactions found that, while men and women show considerable overlap, consistent sex differences do emerge (Herzog 2007). Women generally exhibit more positive attitudes and stronger support for animal welfare, whereas men are more commonly associated with practices such as hunting, exploitation, or abuse. Effect sizes of a sex difference vary by domain, ranging from small (e.g., attachment) to large (e.g., animal rights activism, abuse).

Heterospecific play interactions reveal intriguing similarities between humans and nonhuman primates. Nonhuman primates often engage other species using play behaviors that resemble their own species-typical patterns. A comparable dynamic is observed in human–pet interactions, where owners frequently attempt to initiate play using human-specific cues. However, those who adopt the communicative signals of the animal – such as species-typical gestures or postures – tend to be more successful (Rooney et al. 2001).

A central question is which types of observed interspecific interactions in non-human primates may qualify as analogous to pet keeping. Sterneberg-Van der Maaten et al. (2016) defined a ‘companion animal’ as any domesticated, captive-bred, or wild-caught species that resides permanently within a human community and is maintained for companionship, enjoyment, work (e.g., guide dogs for the visually impaired, hearing dogs, police or military dogs), or psychological support. This definition, which encompasses species such as dogs, cats, horses, rabbits, ferrets, guinea pigs, reptiles, birds, and ornamental fish, emphasizes the enduring and long-term nature of these human-animal relationships. The clearest candidates are thus cases of cross-species adoption, where individuals not only tolerate or briefly interact with heterospecifics, but actively care for and maintain close proximity to them. One particularly compelling case is described by (Izar et al. 2006), in which a wild infant common marmoset (*Callithrix jacchus*) was adopted by a group of wild black-striped capuchin monkeys (*Sapajus libidinosus*) in Brazil. The marmoset remained with the group for at least ten months and was well integrated socially, receiving nurturant care from two successive adoptive “mothers” and broad tolerance from other group members. Similarly, in a more anthropogenic context, a subadult crested macaque (*Macaca nigra*) in an Israeli zoo was reported to have adopted a chicken that had wandered into its enclosure, engaging in affiliative and protective behaviors toward the bird (ABC News 2017). Another context in which pet-keeping analogs may arise is among primates living in close association with humans. The most well-known example is that of Koko, the western gorilla (*Gorilla gorilla gorilla*), who formed a close bond with a kitten named All Ball. Koko displayed maternal behaviors such as cradling, grooming, and protective posturing, treating the kitten as a surrogate infant in the absence of conspecific offspring (Patterson 1985). Together, these examples raise the possibility that some behavioral tendencies underlying human pet keeping, such as extended caregiving and emotional bonding toward heterospecifics, may have antecedents in the heterospecific social behavior of some non-human primates.

### Limitations

Several limitations should be considered when interpreting our findings. First, our dataset is heterogeneous in origin, comprising the peer-reviewed literature, media reports, and survey responses, each with varying levels of observational detail and reliability. This introduces potential biases, including uneven reporting across species, contexts, and behavior types, as well as a likely overrepresentation of more striking or anthropomorphically appealing interactions (e.g., play). In particular, observations derived from public media may preferentially capture unusual, visually striking, or human-like interactions and should therefore not be taken as representative of the natural frequency of these behaviors. Extracting data on the frequency and duration of interactions was particularly challenging, as such information is often not reported. Additionally, zoo-based interactions are likely underrepresented, since our survey targeted primarily field primatologists, although we did include relevant cases from the literature. Sexual behaviors were also likely underreported, as we did not systematically include data on interspecific hybrids, which could have provided indirect evidence of cross-species mating. As such, our findings provide a first exploratory synthesis of heterospecific affiliative behavior in primates and a framework for generating future hypotheses and comparative analyses. Consequently, the present study is best viewed as a conceptual and hypothesis-generating framework rather than a systematic estimate of the prevalence or evolutionary significance of heterospecific affiliative behavior in primates.

### Future research directions

Our findings underscore the importance of more consistent documentation of heterospecific social interactions, particularly rare affiliative or socio-positive behaviors that are often overlooked in conventional doctoral research or long-term field studies. These interactions, though infrequent, may provide critical insights into the social flexibility, cognitive capacities, and ecological adaptations of primates. We recommend incorporating dedicated protocols for documenting such events into long-term behavioral monitoring programs and encouraging the publication of well-substantiated anecdotal observations in appropriate outlets.

One pattern emerging from previous research is that affiliative interspecific interactions such as grooming and play appear more common in species facing minimal interspecific competition, particularly those with little dietary overlap (Struhsaker 1981; Fimbel 1992). This hypothesis merits systematic testing across primate taxa and ecosystems to assess how ecological niche differentiation influences the frequency and nature of heterospecific affiliation. To test this, it is important to study each species niche at the same location, and niches, particularly diet, can vary substantially over short distances (Chapman et al. 2002). Furthermore, non-agonistic interspecific interactions may be more frequent during periods of resource abundance, when reduced competition and lower ecological stress make affiliative or tolerant interspecific encounters more likely (Freymann et al. 2023). Under these conditions, primates may have less incentive to act agonistically toward potential heterospecific competitors, as the immediate costs of sharing space or resources are diminished.

Another avenue for future research is the extent to which primates engage socially with species they also prey upon. While some studies suggest that primates preferentially direct social behavior toward non-prey species, this is not consistently the case. For example, lion-tailed macaques (*Macaca silenus*) have been observed grooming an Indian giant squirrel (*Ratufa indica*), a known prey species (Kumara and Mewa Singh cited in Vasava et al. 2013), and chimpanzees have been documented engaging socially with red-tailed monkeys and blue monkeys (*Cercopithecus mitis*), both of which are occasional prey (Freymann et al. 2023). These examples challenge the assumption that predation risk excludes affiliative engagement and suggest a need for more nuanced, context-dependent analyses.

Emerging evidence also suggests that solitary or socially marginalized individuals may be particularly likely to engage in heterospecific social behavior (Waser 1987; Fleury & Gautier-Hion 1997; Gathua 2000). For instance, solitary male red-tailed monkeys in Kibale received a disproportionately high share of grooming from ashy red colobus (*Piliocolobus tephrosceles*) despite limited association frequencies (Struhsaker 1981). Similar patterns were observed in solitary blue monkeys and grey-cheeked mangabeys (*Lophocebus albigena*) (ibid.). These findings raise the possibility that solitary individuals who lack conspecific social partners may turn to heterospecifics for social buffering, analogous to the role pets can play in mitigating psychosocial stress in humans (reviewed in Serpell and Paul 2011).

The “learning to mother” hypothesis (Lancaster 1971; Hrdy 1976) proposes that handling other conspecifics’ infants allows subadults, especially females, to acquire parenting skills that enhance future offspring survival. Although our dataset lacked sufficient specific age data to test this directly for heterospecifics, future studies should address this with more detailed demographic information. This hypothesis has also been extended to human pet keeping. According to Serpell & Paul (2011), the “parenting experience” hypothesis predicts that interest in pet keeping should peak in preadolescent and adolescent females, who stand to benefit most from gaining nurturing experience. Supporting this, research shows that in western countries children’s interest in pets is generally strongest among girls aged 8–12, particularly those without younger siblings, and typically declines thereafter (Paul and Serpell 1992; Melson et al. 1997).

Interspecific interactions may be influenced by physical and behavioral compatibility. Brooks and Burghardt (2023) argue that differences in preferred play behavior and morphology between species can make such interactions difficult to sustain. This suggests that similar size and strength between partners may increase the likelihood of interspecific play, particularly among juveniles or subadults. In addition to physical compatibility, future work should examine how behavioral cues, posture, or responsiveness influence recipient selection in affiliative interspecific contexts. Such cues may be key in facilitating or inhibiting interaction across species boundaries.

The behavioral repertoire of heterospecific social interactions is likely broader than typically recognized. It includes rare but significant behaviors such as coalitionary predator mobbing, documented, for example, between Bornean white-bearded gibbons (*Hylobates albibarbis*) and maroon langurs (*Presbytis rubicunda*) (Wilcox et al. 2016). Including these events in systematic data collection will help capture the full spectrum of cross-species interactions. In particular, subtle and short-lived interactions with insects and other small-bodied taxa are likely substantially underreported. Such behaviors may occur relatively frequently in both wild and captive settings but are often inconspicuous, difficult to interpret, and less likely to be systematically documented than interactions involving mammals or birds. Future studies would benefit from explicitly incorporating protocols for recording brief or low-salience heterospecific interactions, which may reveal a broader spectrum of exploratory or affiliative behavior than is currently recognized.

It is plausible that some forms of heterospecific affiliation are culturally inherited. In white-faced capuchins, allospecific grooming has been largely confined to current or former members of a single group at one site, suggesting a socially transmitted tradition (Perry et al. 2003; Rose et al. 2003). More recently, Goldsborough et al. (2025) described a striking cultural tradition in which immature male white-faced capuchins repeatedly abducted infant mantled howler monkeys, a behavior that spread within the group despite its apparent lack of adaptive function. Such cases echo arguments in human ethology: Herzog (2014) contends that the wide cultural variation in pet-keeping practices (Gray and Young 2011), along with rapid shifts in trends (such as the changing popularity of dog breeds in the United States), suggests that pet-keeping is primarily shaped by social learning and imitation-driven cultural evolution.

At the individual level, however, heterospecific social interactions need not reflect socially transmitted traditions. Behaviors that are rare, idiosyncratic, or consistently restricted to one or two individuals within a population are more parsimoniously interpreted as expressions of stable individual differences, such as personality traits or behavioral predispositions (e.g., elevated exploratory tendency or neophilia; Réale et al. 2010; Nord et al. 2022). Attention to actor identity is therefore critical for distinguishing population-level cultural phenomena from individual-specific pathways to heterospecific affiliation.

## Conclusions

We provide the first systematic synthesis of interspecific social interactions involving nonhuman primates, revealing a surprising diversity of social behaviors directed toward heterospecifics in both wild and captive contexts. While most interactions were initiated by primates and were overwhelmingly affiliative in nature (particularly play and grooming) our findings also highlight rare but notable cases of abuse, as well as complex interactions resembling cross-species adoption or proto–pet keeping. Patterns of interaction were shaped by factors such as age, sex, and species identity, with adult females showing the strongest tendency toward affiliative behavior and juveniles more likely to engage in play. Although constrained by uneven reporting and limited behavioral detail, our findings suggest that certain forms of affiliative behavior, such as grooming, caregiving, and exploratory play, can extend across species boundaries under some ecological and social conditions. These behaviors may provide a useful comparative framework for considering possible evolutionary precursors to aspects of human–animal companionship. However, most heterospecific social interactions involving primates appear to be rather ephemeral in nature, in contrast to pet-keeping in humans, which typically entails long-term companionship.

These observations have several broader implications. First, as Brooks and Burghardt (2023) argue, heterospecific interactions often involve the ability to read and respond appropriately to the social cues of other species that are sometimes taxonomically distant, highlighting important links between perception, communication, and social cognition. Second, understanding patterns of interspecific affiliation and aggression can inform captive management strategies. Knowing which species are more likely to engage in cooperative rather than competitive interactions may help guide decisions about housing animals in mixed-species enclosures, improving both welfare and enrichment. Third, these findings contribute to comparative studies of social evolution by revealing that complex social behaviors like play, grooming, and caregiving are not always constrained by species boundaries, and may emerge under specific ecological or developmental conditions. Fourth, interspecific social interactions have implications for disease ecology. Close physical contact across species lines, whether in wild or captive settings, can increase the risk of cross-species pathogen transmission, with potential consequences for wildlife health and conservation. Finally, as habitat loss and fragmentation bring wildlife into closer contact with humans and domesticated animals, understanding the dynamics of heterospecific affiliation may help anticipate novel forms of interspecies interaction (and conflict) at the human–wildlife interface.

## Supplementary Information

Below is the link to the electronic supplementary material.

Supplementary file1 (XLSX 54 KB)

Supplementary file2 (DOCX 23 KB)

## Data Availability

All data supporting the findings of this study are provided in the Supplementary Information accompanying this article.
