# Supplementary material for: Heterospecific social behavior in primates: insights into the evolutionary roots of pet-keeping
Source: Primates. 2026 Jul 21;67(6):629–46. doi: 10.1007/s10329-026-01281-0 (PMC13731472; doi:10.1007/s10329-026-01281-0)
Supplement: Supplementary file 1 [file 10329_2026_1281_MOESM2_ESM.docx]

**Table S2**: Most frequent interspecific social interactions observed *under human care*, showing dyads with two or more recorded events. The table lists the primary species (typically a primate), the associated other species, and the frequency of observed interactions.

| **Primary species** | **Other species** | **Frequency** |
| --- | --- | --- |
| *Theropithecus gelada* | *Ammotragus lervia* | 5 |
| *Ateles sp.* | *Cebus sp.* | 4 |
| *Papio hamadryas* | *Loxodonta africana* | 4 |
| *Lemur catta* | *Eulemur rufus* | 4 |
| *Pongo abelii* | *Symphalangus syndactylus* | 4 |
| *Saguinus labiatus* | *Leontocebus weddelli* | 4 |
| *Saguinus mystax* | *Leontocebus nigrifrons* | 4 |
| *Cercocebus atys* | *Cercocebus torquatus* | 3 |
| *Macaca nigra* | *Gallus gallus domesticus* | 3 |
| *Lemur catta* | *Eulemur macaco* | 3 |
| *Papio sp.* | *Phacochoerus africanus* | 3 |
| *Gorilla gorilla* | *Felis catus* | 3 |
| *Pongo sp.* | *Canis familiaris* | 3 |
| *Semnopithecus entellus* | *Cervus duvaucelii* | 2 |
| *Cebuella pygmaea* | *Saguinus midas* | 2 |
| *Macaca sylvanus* | *Ammotragus lervia* | 2 |
| *Macaca fuscata* | *Ammotragus lervia* | 2 |
| *Macaca mulatta* | *Canis familiaris* | 2 |
| *Papio sp.* | *Felis catus* | 2 |
| *Saimiri sciureus* | *Felis catus* | 2 |
| *Pongo pygmaeus* | *Macaca silenus* | 2 |

**Table S3**: Most frequent interspecific social interactions observed *in the wild*, showing dyads with two or more recorded events. The table lists the primary species (typically a primate), the associated other species, and the frequency of observed interactions.

| **Primary species** | **Other species** | **Frequency** |
| --- | --- | --- |
| *Cebus imitator* | *Ateles geoffroyi* | 19 |
| *Macaca fuscata* | *Cervus nippon* | 12 |
| *Pan troglodytes* | *Cercopithecus ascanius* | 11 |
| *Macaca mulatta* | *Canis familiaris* | 11 |
| *Piliocolobus tephrosceles* | *Lophocebus albigena* | 8 |
| *Pan troglodytes* | *Dendrohyrax dorsalis* | 6 |
| *Macaca fascicularis* | *Presbytis femoralis* | 6 |
| *Lophocebus albigena* | *Cercopithecus ascanius* | 6 |
| *Piliocolobus tephrosceles* | *Cercopithecus ascanius* | 6 |
| *Cercopithecus mitis* | *Cercopithecus ascanius* | 6 |
| *Pan paniscus* | *Cercopithecus ascanius* | 5 |
| *Lophocebus albigena* | *Piliocolobus tephrosceles* | 5 |
| *Piliocolobus tephrosceles* | *Cercopithecus mitis* | 5 |
| *Hoolock hoolock* | *Trachypithecus phayrei* | 5 |
| *Sapajus apella* | *Nasua nasua* | 4 |
| *Papio anubis* | *Theropithecus gelada* | 4 |
| *Papio hamadryas* | *Canis familiaris* | 4 |
| *Trachypithecus johnii* | *Semnopithecus priam* | 4 |
| *Piliocolobus badius* | *Colobus polykomos* | 4 |
| *Lemur catta* | *Hapalemur meridionalis* | 4 |
| *Papio ursinus* | *Panthera leo* | 4 |
| *Sapajus libidinosus* | *Callithrix jacchus* | 4 |

**Table S4.** Frequency of interspecific social interactions categorized by the family of the primary species (typically a primate) and the family of the other species involved. Counts represent the number of recorded interactions for each unique family pairing. Data from *both captivity and the wild*.

| **Family primary species** | **Family other species** | **Count** |
| --- | --- | --- |
| Aotidae | Dasyproctidae | 1 |
| Aotidae | Megalonychidae | 1 |
| Atelidae | Atelidae | 5 |
| Atelidae | Cebidae | 8 |
| Atelidae | Cracidae | 1 |
| Atelidae | Megalonychidae | 1 |
| Atelidae | Myrmecophagidae | 1 |
| Atelidae | Pitheciidae | 1 |
| Atelidae | Procyonidae | 2 |
| Atelidae | Sciuridae | 1 |
| Callitrichidae | Callitrichidae | 23 |
| Callitrichidae | Canidae | 1 |
| Callitrichidae | Dasyproctidae | 1 |
| Callitrichidae | Pitheciidae | 2 |
| Callitrichidae | Psittacidae | 1 |
| Callitrichidae | Sciuridae | 1 |
| Cebidae | Atelidae | 24 |
| Cebidae | Callitrichidae | 8 |
| Cebidae | Canidae | 4 |
| Cebidae | Cebidae | 2 |
| Cebidae | Erethizontidae | 1 |
| Cebidae | Felidae | 2 |
| Cebidae | Procyonidae | 4 |
| Cebidae | Psophiidae | 1 |
| Cercopithecidae | Bovidae | 17 |
| Cercopithecidae | Bufonidae | 3 |
| Cercopithecidae | Canidae | 28 |
| Cercopithecidae | Cercopithecidae | 99 |
| Cercopithecidae | Cervidae | 18 |
| Cercopithecidae | Corvidae | 1 |
| Cercopithecidae | Elephantidae | 4 |
| Cercopithecidae | Felidae | 9 |
| Cercopithecidae | Galagidae | 2 |
| Cercopithecidae | Herpestidae | 2 |
| Cercopithecidae | Hominidae | 5 |
| Cercopithecidae | Hylobatidae | 3 |
| Cercopithecidae | Lorisidae | 1 |
| Cercopithecidae | Paguroidea | 1 |
| Cercopithecidae | Phasianidae | 4 |
| Cercopithecidae | Sciuridae | 5 |
| Cercopithecidae | Suidae | 5 |
| Cercopithecidae | Tarsiidae | 1 |
| Galagidae | Canidae | 1 |
| Hominidae | Anomaluridae | 1 |
| Hominidae | Ardeidae | 1 |
| Hominidae | Bovidae | 3 |
| Hominidae | Bufonidae | 1 |
| Hominidae | Canidae | 6 |
| Hominidae | Cercopithecidae | 24 |
| Hominidae | Felidae | 5 |
| Hominidae | Galagidae | 1 |
| Hominidae | Herpestidae | 3 |
| Hominidae | Hominidae | 4 |
| Hominidae | Hylobatidae | 4 |
| Hominidae | Lacertidae | 1 |
| Hominidae | Leporidae | 1 |
| Hominidae | Lorisidae | 1 |
| Hominidae | Manidae | 4 |
| Hominidae | Musophagidae | 1 |
| Hominidae | Mustelidae | 2 |
| Hominidae | Procaviidae | 6 |
| Hominidae | Sciuridae | 5 |
| Hominidae | Strigidae | 2 |
| Hominidae | Suidae | 3 |
| Hominidae | Viverridae | 2 |
| Hylobatidae | Cercopithecidae | 5 |
| Hylobatidae | Hominidae | 1 |
| Hylobatidae | Muridae | 1 |
| Hylobatidae | Tapiridae | 1 |
| Indriidae | Lemuridae | 1 |
| Lemuridae | Cuculidae | 1 |
| Lemuridae | Lemuridae | 16 |
| Lorisidae | Cercopithecidae | 1 |
| Lorisidae | Sciuridae | 1 |
| Pitheciidae | Cebidae | 1 |
